# Supplementary material for: Diagnostic Efficacy of Serum Asialo α1-Acid Glycoprotein Levels for Advanced Liver Fibrosis and Cirrhosis in Patients with Chronic Hepatitis B Compared to That in Healthy Subjects: A Prospective Study
Source: J Clin Med. 2023 Jan 16;12(2):712. doi: 10.3390/jcm12020712 (PMC9863335; doi:10.3390/jcm12020712)
Supplement: Supplementary file 1 [file jcm-12-00712-s001.zip › jcm-2100422-supplementary.pdf]

Table S1. The diagnostic performance of serum AsAGP according to fibrosis stages of CHB patients comparing healthy control.

	CHB with F0-1 versus. Healthy control	CHB with F2 versus healthy control	CHB with F3 versus healthy control
AUC (95% CI)	0.610 (0.503 - 0.717)	0.513 (0.342 - 0.684)	0.685 (0.562 – 0.809)
Optimal cut-off (µg/ml)	0.896	1.001	0.926
Sensitivity	84.6%	62.5%	82.6%
Specificity	50%	60.3%	54.4%

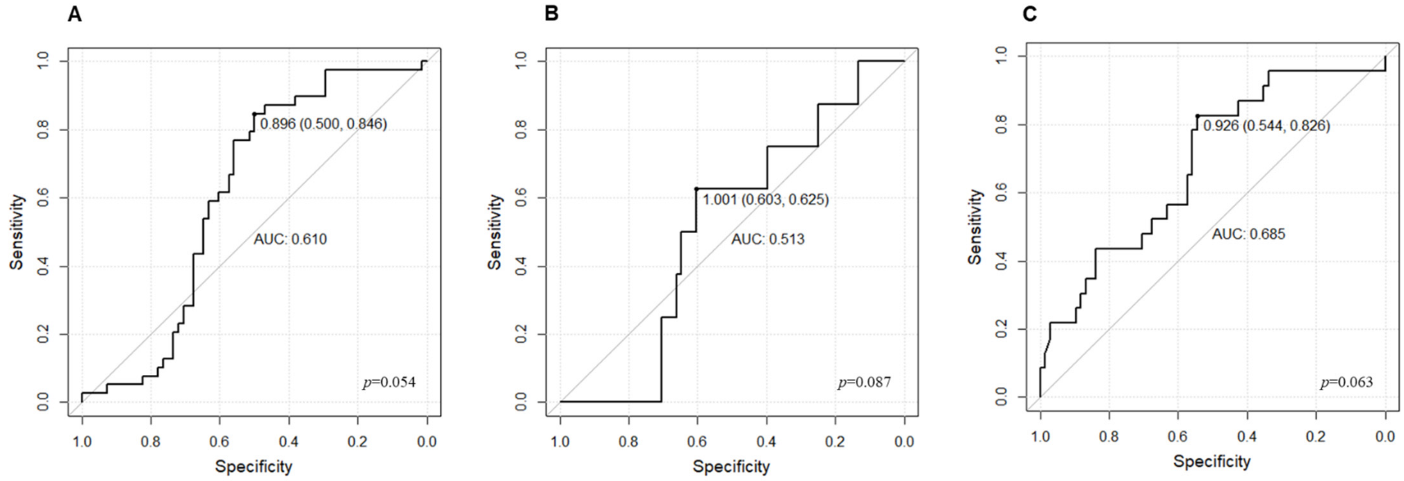

Figure S1. The AUROC of serum AsAGP level for differentiating liver fibrosis stages in CHB patients comparing healthy control. A; CHB F0-1 versus healthy control, B; CHB F2 versus healthy control, C; CHB F3 versus healthy control.

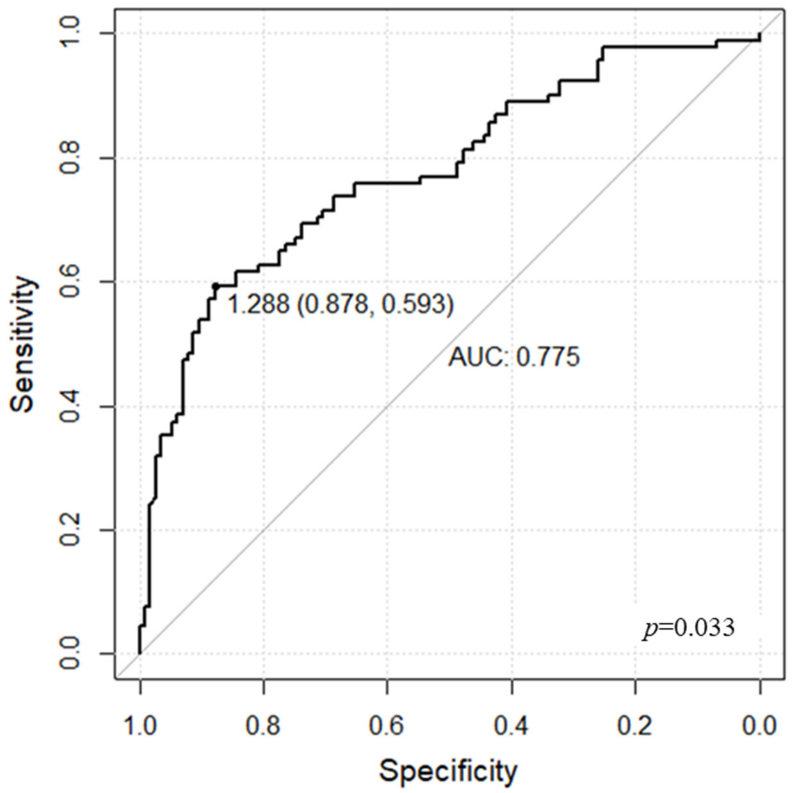

Figure 2. The AUROC of serum AsAGP level for differentiating advanced fibrosis in CHB patients comparing healthy control.
